# The epigenetic players and the chromatin marks involved in the articular cartilage during osteoarthritis

**DOI:** 10.3389/fphys.2023.1070241

**Published:** 2023-01-17

**Authors:** Jérôme E. Lafont, Sherine Moustaghfir, Anne-Laure Durand, Frédéric Mallein-Gerin

**Affiliations:** Laboratoire de Biologie Tissulaire et Ingénierie Thérapeutique CNRS—UMR 5305, Université Claude Bernard Lyon1, University of Lyon, Lyon, France

**Keywords:** cartilage, histone and DNA modifications, gene regulation, osteoarthritis, inflammation

## Abstract

Epigenetics defines the modifications of the genome that do not involve a change in the nucleotide sequence of DNA. These modifications constitute a mechanism of gene regulation poorly explored in the context of cartilage physiology. They are now intensively studied by the scientific community working on articular cartilage and its related pathology such as osteoarthritis. Indeed, epigenetic regulations can control the expression of crucial gene in the chondrocytes, the only resident cells of cartilage. Some epigenetic changes are considered as a possible cause of the abnormal gene expression and the subsequent alteration of the chondrocyte phenotype (hypertrophy, proliferation, senescence…) as observed in osteoarthritic cartilage. Osteoarthritis is a joint pathology, which results in impaired extracellular matrix homeostasis and leads ultimately to the progressive destruction of cartilage. To date, there is no pharmacological treatment and the exact causes have yet to be defined. Given that the epigenetic modifying enzymes can be controlled by pharmacological inhibitors, it is thus crucial to describe the epigenetic marks that enable the normal expression of extracellular matrix encoding genes, and those associated with the abnormal gene expression such as degradative enzyme or inflammatory cytokines encoding genes. In this review, only the DNA methylation and histone modifications will be detailed with regard to normal and osteoarthritic cartilage. Although frequently referred as epigenetic mechanisms, the regulatory mechanisms involving microRNAs will not be discussed. Altogether, this review will show how this nascent field influences our understanding of the pathogenesis of OA in terms of diagnosis and how controlling the epigenetic marks can help defining epigenetic therapies.

## 1 Introduction

The human genome has a double helix DNA structure of 2 m length protected by the nucleus of only a few micrometers length. Therefore, the DNA is extremely compacted and integrated into a nucleoprotein structure called chromatin (Göndör, 2016). The chromatin corresponds to the complex of DNA with the histone proteins, which are small structural proteins of 11–14 kDa. The structure of chromatin is highly dynamic and remains accessible to the transcriptional machinery, allowing a highly coordinated gene expression. One state of compaction, the euchromatin, which accounts for 80%–90% of nuclear DNA, contains the majority of genes. This region is molecularly relaxed and is therefore potentially more transcriptionally active. The second state of compaction, the heterochromatin, contains 10%–20% of the nucleus’s DNA, and corresponds to a condensed and transcriptionally inactive chromatin. It is located mainly on the periphery of the nucleus. There is a distinction between constitutive heterochromatin that responds to never-transcribed chromosomal regions, such as telomeres, and optional heterochromatin, which corresponds to repressed (or not) regions depending on cell type, such as genes involved in cell differentiation. The active or repressed state of a gene is therefore influenced by its location in euchromatin or heterochromatin. The chromatin may undergo epigenetic changes in a spatio-temporal way that governs its degree of compaction.

The term epigenetics defines all the changes that occur at the genome level, by modulating the gene transcription (Bernstein et al., 2007). These changes, which are communicable, dynamic and reversible, are influenced by the environment, that is, by the signals the cell receives. They are identified as biochemical marks, and are catalyzed by specific enzymes at the DNA or histones level. It is therefore the epigenetic mechanisms that orchestrate a differential reading of genes over the course of life and according to cell type.

These modifications constitute a mechanism of gene regulation poorly explored in the context of cartilage physiology. They are now more studied by the scientific community working on articular cartilage and its related pathology such as osteoarthritis. Indeed, epigenetic regulations can control the expression of crucial gene in the chondrocytes. Some epigenetic changes are considered as possible cause of the abnormal gene expression and the subsequent alteration of the chondrocyte phenotype (hypertrophy, proliferation, senescence…). A better knowledge of the epigenetic signature of the joint tissues can help understanding the underlying mechanisms of osteoarthritis or finding new therapeutic strategies. Osteoarthritis is the most common disease of the joints. It affects one or more weight-bearing or non-weight-bearing joints, including those of the hand, knee, intervertebral discs, and hip. It results from a degradation of cartilage that can lead to severe pain and loss of mobility. With the aging population, osteoarthritis is becoming a major public health problem. The incidence and prevalence of osteoarthritis increased by two and ten times between the ages of 30 and 65 and continued to increase thereafter (Felson et al., 2000). After age 65, 60% of the population has signs of osteoarthritis of the hand, 33% of osteoarthritis of the knee and 5% of osteoarthritis of the hip (Martel-Pelletier et al., 2016). As an example, it has been estimated that in 2030, 20% of the population would suffer from osteoarthritis in the United States (Bhatia et al., 2013). Although highly associated with age, traumatic joint injuries also contribute to the development of osteoarthritis. Indeed, 12% of all cases of osteoarthritis are the result of a hip, ankle or knee injury (Brown et al., 2006). There are also other risk factors, such as obesity, genetics, mechanical stress, mild systemic inflammation or certain metabolic diseases (Stiebel et al., 2014; Kluzek et al., 2015).

Osteoarthritis is now considered as a whole joint disease, involving the cartilage, the subchondral bone, the synovial membrane and the ligaments (Loeser et al., 2012). Histology of the joint tissues primarily reveals the presence of fibrillations on the articular surface, which may progress to deeper fissures (Sulzbacher, 2013). This process is then accompanied by the progressive erosion of the cartilage ECM and the subchondral bone cracks. In parallel, many cells and tissue alterations are observed: Cartilage calcification, vascular invasion (Fuerst et al., 2009; Burr and Gallant, 2012), bone remodeling (Mahjoub et al., 2012), and inflammation of the synovial membrane (Goldring and Otero, 2011). Finally, the chondrocytes are exposed to abnormal signals, react in different ways, such as apoptosis, proliferation and aberrant phenotype (hypertrophic or dedifferentiated) (Sandell and Aigner, 2001; Lotz et al., 2010). The overall activity of chondrocytes is impaired, with a decreased anabolism closely associated with an increased catabolism. The initiating events of osteoarthritis are unclear and a better understanding of the intrinsic mechanisms occurring in the chondrocyte, notably at the epigenetic level, could help finding new therapeutic targets.

**FIGURE 1:**
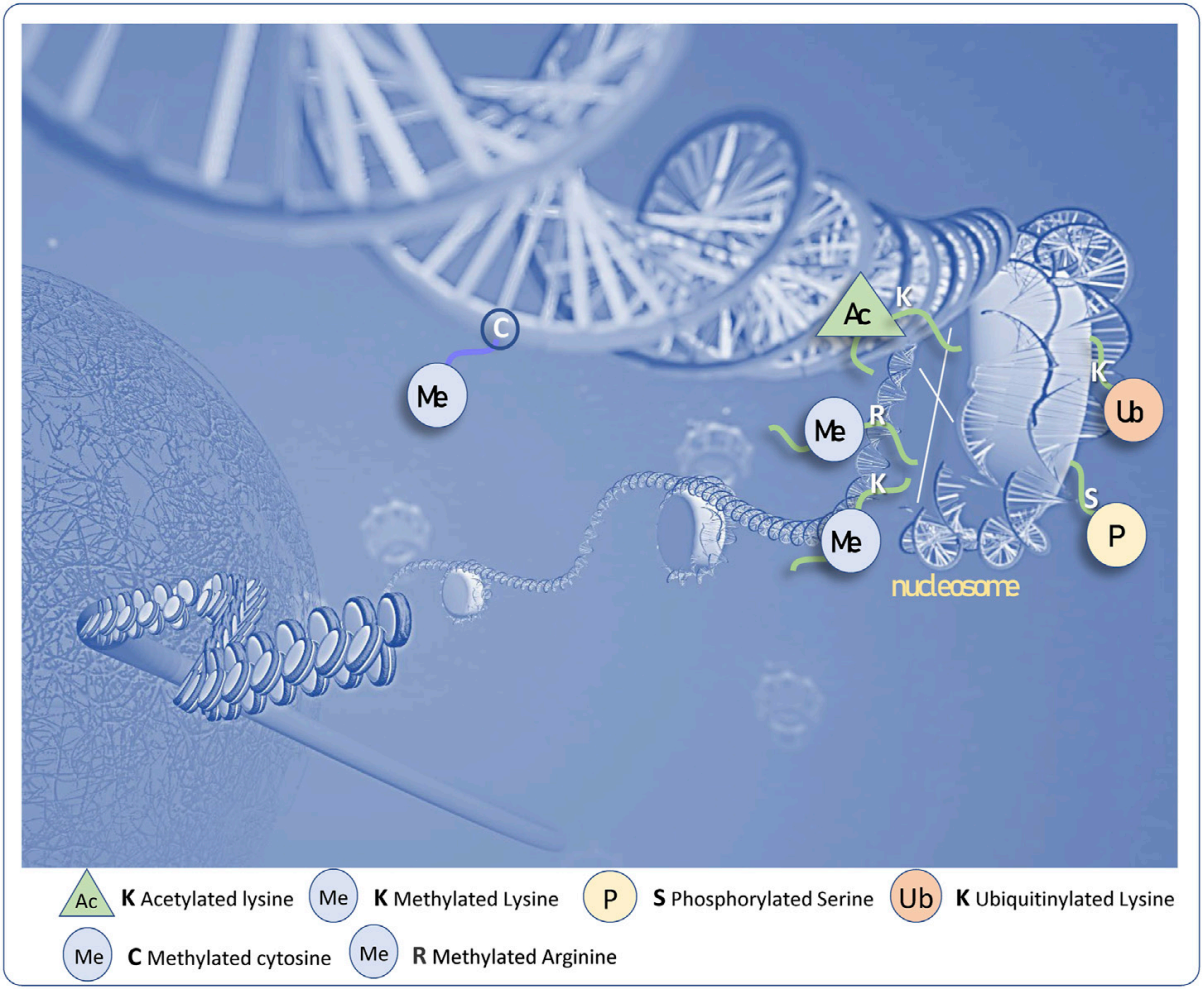
The multiple epigenetic marks associated to the DNA and histones.

The review mainly deals with the progress of two categories of epigenetic modifications occurring on the chromatin, and more specifically in the context of cartilage pathophysiology (Figure 1): First, we detail the DNA methylations which are associated with OA and notably those described in the extracellular matrix or inflammatory encoding genes. We aim to draw the most comprehensive DNA profile of OA samples with more attention paid to the nature of each analysed tissue. Second, we detail the main histone modifications, with a focus on acetylation and methylation marks observed in the articular cartilage. Then we discuss how this knowledge influences our understanding of the pathogenesis of OA and to what extent changing the histone marks can be a valuable strategy to prevent OA.

### 1.1 DNA methylation

DNA methylation, which is the most studied epigenetic mechanism, involves the addition of a methyl group (CH3) in carbon 5 of a cytosine to form 5-methylcytosine (5 mC) (Bernstein et al., 2007). This covalent modification does not alter the DNA sequence but adds a mark allowing a differential reading of the genome (Jeltsch and Jurkowska, 2014). In mammals, DNA methylation is mainly performed at a CpG dinucleotide, when cytosine is linked by a phosphodiester bridge to a guanine. Generally, DNA methylation of a promoter region is closely associated with gene suppression. It has been well described in the X-chromosome inactivation process (Sharp et al., 2011; Cotton et al., 2015).

DNA methylation is a reversible process catalyzed by methyltransferase DNA (DNMT), whose cofactor, a methyl donor, is S-Adenosylmethionine (SAM). In eukaryotes, the DNMT family consists of five members identified by sequence homology: DNMT1, DNMT2, DNMT3A, DNMT3B, and DNMT3L. However, only the proteins DNMT1, DNMT3A and DNMT3B possess methyltransferase DNA activity. They act in a coordinated way to establish the DNA methylation profile. DNMT1 is described as the only maintenance methyltransferase (allowing a given methylation profile to be maintained along the time), while the DNMT3A and DNMT3B are *de novo* methyltransferases (occurring on unmethylated DNA in undifferentiated cells and establishing a new pattern of methylation) (Jeltsch and Jurkowska, 2014)*.*

#### 1.1.1 DNA methylation profile

##### 1.1.1.1 The CpG islands vs. regions poor in CpG

In the human genome, 70%–80% of CpG dinucleotides are methylated (Ehrlich et al., 1982). These CpG sites are distributed non-uniformly along the genome. Overall, the frequency of CpG dinucleotides is relatively low (about 1%). In the human genome, it has been reported that nearly 60% of genes have a CpG island within their promoter region (Bernstein et al., 2007). Overall, these regions are generally hypomethylated, i.e., low methylated, whether the gene is suppressed or not.

The surrounding regions of the CpG islets are poorer in CpG sites and are classified into several categories according to their location “CpG island shores” (up to 2 kb spanning region), “CpG island shelves” (from to 2–4 kb spanning region), and “open sea” (more than 4 kb spanning region), and have a level of methylation depending on the cell type or tissue.

#### 1.1.2 Regulatory function of DNA methylation

##### 1.1.2.1 At the promoter level

The effect of DNA methylation on gene expression has been extensively studied in gene-promoter regions. It has been shown that an increase in the level of DNA methylation is closely associated with gene suppression. This negative correlation appears to be based on two complementary mechanisms. On the one hand, the presence of methylated cytosines can directly prevent the binding of transcription factors on their targets (Mancini et al., 1999; Bell and Felsenfeld, 2000; Hark et al., 2000). On the other hand, methylated CpGs are recognized by at least three protein families: Methyl-Binding domain (MBD) proteins, zinc finger proteins and SRA (SET and RING-Associated) proteins, which are able to reshape chromatin where CpGs are methylated, thus making heterochromatin inaccessible to transcription (Ballestar and Wolffe, 2001; Parry and Clarke, 2011)^.^

##### 1.1.2.2 At the level of intragenic regions

The negative correlation between gene expression and methylation of a promoter cannot be transferred to other genomic regions. Several studies have also reported a positive and paradoxically negative correlation between transcription activation and DNA methylation in the body of genes.

In recent years, other roles of DNA methylation at the body level of genes have been highlighted. For example, it has been shown that DNA methylation is capable of regulating the activation of alternative promoters, the alternative splicing, the activity of activating sequences (also known as enhancers), and finally, the transcription of non-coding RNAs (nCNVs) (Kulis et al., 2013).

##### 1.1.2.3 At the level of intergenic regions

The intergenic regions are highly methylated but the role of this epigenetic modification is still poorly understood. It is proposed, however, that DNA methylation should be involved, on the one hand, in maintaining the integrity, and in the stability of the genome (by inactivating repeated elements) on the other hand (Jones, 2012).

### 1.2 DNA methylation in osteoarthritis

#### 1.2.1 The “candidate gene” strategy

##### 1.2.1.1 Genes contributing to osteoarthritis

Given the important role of DNA methylation in many pathologies, several teams have evaluated the contribution of the DNA methylation of cytosine in osteoarthritis. In 2005, a study provided the first evidence of an association between changes in the level of DNA methylation and the level of expression of several genes in osteoarthritic cartilage (Roach et al., 2005). Indeed, this study reports a demethylation in OA cartilage, of certain CpG sites located in the promoter of the genes encoding *MMP-3*, *MMP-9*, *MMP-13*, and *ADAMTS-4* that correlates with their increased expression. Subsequently, two studies have identified the mechanism involved in controlling *MMP-13* expression through modulation of the DNA methylation level. Demethylation of the CpG site, located −110 bp from the TSS (Transcription Start Site) of this gene, has been shown to facilitate the access and binding of transcription factors CREB and HIF-2α (Bui et al., 2012; Hashimoto et al., 2013). Thus, the increase in recruitment of CREB and HIF-2α on the promoter of *MMP-13* in OA chondrocytes contributes to the increased expression of this MMP in osteoarthritis. Such a mechanism has also been shown for the *SOST* gene, a Wnt pathway inhibitor, whose hypomethylation of the promoter in OA chondrocytes, facilitates the recruitment of Smad 1/5/8, and therefore stimulates its expression (Papathanasiou et al., 2015). Interestingly, changes in the level of DNA methylation of this gene have also been reported in human bone (Lhaneche et al., 2016) indicating an important role of DNA methylation in controlling *SOST* expression.

##### 1.2.1.2 Genes contributing to the synthesis of a cartilaginous matrix

Similarly, DNA methylation influences the expression of genes that contribute to anabolism during osteoarthritis. Methylation levels of the promoter of *COL9A1* have been shown to be higher in OA chondrocytes compared to normal chondrocytes. This hypermethylation leads to a decrease in the recruitment of Sox9 on the promoter of this gene (Imagawa et al., 2014). Thus, the induction of the *COL9A1* gene by Sox9 is lower in osteoarthritic chondrocytes. In addition, methylation of the Sox9 promoter has been shown to be elevated in OA cartilage compared to normal cartilage, resulting in reduced recruitment of transcription factors such as CREB (Kim et al., 2013). However, the expression of aggrecan and type II collagen does not appear to be modulated through changes of DNA methylations (Poschl et al., 2004; Imagawa et al., 2014).

#### 1.2.2 Effect of stimuli on DNA methylation

External stimuli have been shown to modulate DNA methylation. In this sense, it has been shown that the treatment of joint chondrocytes with pro-inflammatory cytokines (with IL-1β, and TNFα-/oncostatine M) results in demethylation of the proximal promoter of *IL-1β* encoding gene (Hashimoto et al., 2009). This demethylation, which occurs specifically on a CpG site located at −299 bp of the TSS, is required for the expression of this interleukin (Hashimoto et al., 2013). Thus, the synthesis of IL-1β is promoted by the demethylation of DNA in an inflammatory context.

DNA methylation therefore appears to play a key role in the imbalance of cartilage homeostasis by altering the expression of anabolic and catabolic genes. The list of all the differentially methylated genes reported in cartilage or OA chondrocytes is in Table 1. The majority of studies on DNA methylation alteration in osteoarthritis focuses on the cartilage tissue of the joint. However, more recent study found hypomethylation of the promoter encoding IL-6 in synovial fibroblasts from patients with osteoarthritis (Yang et al., 2017). This modification of DNA methylation could contribute to the increased production of IL-6 by synovial fibroblasts. Thus, it appears that the entire joint (not only the cartilage) is subject to alterations in DNA methylation during osteoarthritis.

**TABLE 1:** Studies analyzing DNA methylation using a “candidate gene” strategy in an osteoarthritis context.

Gene	Methylation during OA	Expression during OA	Joint	Demethyl agent	Luciferase vector	References
*ADAMTS-4*	Down	Up	Hip	No	No	Roach et al. (2005)
*DIO2*	Up	Up	Hip and Knee	Yes	No	Bomer et al. (2015)
*GDF5*	Down	Up	Hip and Knee	Yes	Yes	Reynard et al. (2011); Reynard et al. (2014)
*IL-1β*	Down	Up	Hip	Yes	Yes	Hashimoto et al. (2013)
*iNOS*	Down	Up	Hip	Yes	Yes	de Andrés et al. (2013); de Andrés et al. (2016)
*IL-8*	Down	Up	Hip	Yes	Yes	Takahashi et al. (2015)
*Leptine*	Down	Up	Knee	Yes	No	Iliopoulos et al. (2007)
*MMP-13*	Down	Up	Hip	Yes	Yes	Roach et al. (2005); Bui et al. (2012); Hashimoto et al. (2013)
*MMP-9*	Down	Up	Hip	No	No	Roach et al. (2005)
*MMP-3*	Down	Up	Hip	No	No	Roach et al. (2005)
*PHLPP1*	Down	Up	Knee	Yes	Yes	Bradley et al. (2015)
*RUNX2*	Down	Up	Hip	Yes	Yes	Takahashi et al. (2017)
*SOD2*	Up	Down	Hip	Yes	No	Scott et al. (2010)
*SOST*	Down	Up	Knee	Yes	No	Papathanasiou et al. (2015)
*Sox9*	Up	Down	Hip	Yes	No	Kim et al. (2013)

The “candidate gene” strategy consists in analyzing the DNA methylation in genes already known to play a role in osteoarthritis. The majority of these studies compared DNA methylation in OA and normal cartilage. A demethylating agent can be used to assess the effect of DNA methylation loss on gene expression. In addition, the differentially methylated region can also be cloned into a luciferase vector (containing no CpG sites) to assess the direct effect of DNA methylation on the promoter’s activity.

#### 1.2.3 Towards an epigenetic profile

The development of high-speed screening tools has enabled the analysis of the genome-wide DNA methylation profile and the very rapid identification of CpG sites that may be of interest in different pathologies. In recent years, several studies have evaluated the signature of DNA methylation in joint cartilage in the context of osteoarthritis. In most cases, DNA methylation was analyzed with the Illumina Human Methylation 450 (Illumina 450 k) chip, which provides the methylation level of 487557 CpG sites distributed along the genome (including intergenic, intragenic regions, and promoters) to provide the most complete overview.

**FIGURE 2:**
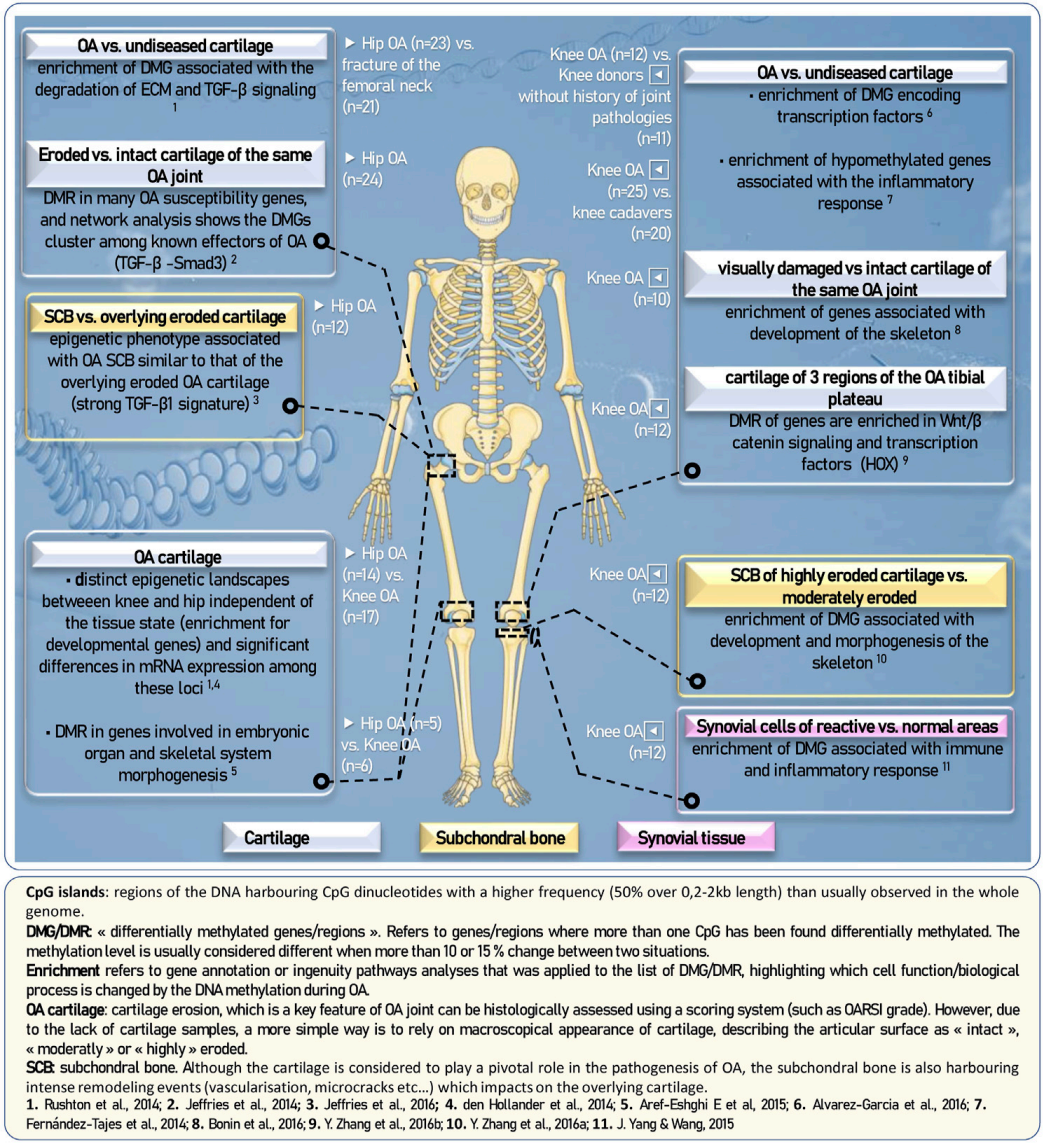
Comparative analyses of DNA methylation profiles of various OA joints.

Comparisons of the methylation profile were made in osteoarthritic cartilage of the knee and hip. Rushton et al. (2014) identified 5,322 cpG sites differentially methylated between these two groups, several of which are located within genes involved in osteoarthritis (including *ADAM12, IL-18, ADAMTS-5, ADAMTS-17),* or in development (*GDF5*, *TGFB2*, *FGFR3*). This result was confirmed by another team, and also revealed an enrichment of HOX genes (involved in the development of the antero-posterior axis) among the differentially methylated (den Hollander et al., 2014). The Table 1 shows the list of DNA methylation profiles of the OA cartilage of the knee and hip, highlighting their similarities and differences. These results suggest that the development of osteoarthritis follows separate signaling pathways depending on the damaged joint (Figure 2).

In addition, several studies have shown that arthritic cartilage samples can be separated into two groups (regarding their methylome) (Fernández-Tajes et al., 2014; Rushton et al., 2014; Alvarez-Garcia et al., 2016). In particular, a subgroup of patients with osteoarthritis of the knee cartilage was defined by an alteration of DNA methylation in the genes associated with the inflammatory and immune response (the second subgroup being associated with TGFβ signaling and cartilage degradation and homeostasis) (Fernández-Tajes et al., 2014; Rushton et al., 2014). A similar result has also been described among patients with hip (Rushton et al., 2014; Rushton et al., 2015). In particular, a population of these patients has been shown to have low levels of methylation along the genes encoding for interleukins IL-2, IL-3, IL-4, and IL-6. Thus, these studies support the existence of an inflammatory phenotype in a subgroup of patients with osteoarthritis.

Several studies have also compared the DNA methylation profile in OA (eroded) or intact cartilage. In all the studies, a number of differentially methylated sites are located in genes known to be associated with osteoarthritis such as *RUNX1*, *RUNX2*, *NFATC1*, *ADAMTS-4*, *COL9A3*, *COL11A2*, and *FGFR2* (Fernández-Tajes et al., 2014; Jeffries et al., 2014; Rushton et al., 2014; Alvarez-Garcia et al., 2016; Zhang et al., 2016a; Bonin et al., 2016). In addition, Gene Ontology’s analysis of differentially methylated genes revealed an enrichment of terms associated with inflammation (Fernández-Tajes et al., 2014; Jeffries et al., 2014) or associated with skeletal (Bonin et al., 2016). These results highlight the existence of an inflammatory component and cartilage remodeling process associated with osteoarthritis. Alvarez-Garcia et al. (2016) identified 45 differentially methylated transcription factors between OA cartilage and normal cartilage. Of these, 10 are also differentially expressed between these two conditions. Thus, changes in DNA methylation along several genes encoding for transcription factors could be an important mechanism in regulating the chondrocytic phenotype in osteoarthritis. A more comprehensive view of these results is presented in Figure 2. This view shows the main comparative studies between OA and control, highlighting which tissue, joint location and stage of OA was used.

Very recently, studies have also shown differences in DNA methylation from the subchondral bone and synovial tissue of patients with osteoarthritis, allowing a more complete DNA methylation profiling of this pathology (Yang and Wang, 2015; Zhang et al., 2016b; Jeffries et al., 2016). This underlines once again the need to take into account for all the tissues that make up the joint to characterize and understand osteoarthritis.

Analysis of the methylation profile of chondrocyte DNA can therefore provide essential data to understand the mechanisms involved in osteoarthritis, or could help defining a pathological cartilage (diagnosis). Although it is accepted that this profile is different depending on the joint and stage of the disease, it is difficult to extend these observations to the entire population and to draw up an epigenetic map. Indeed, any epigenetic variability could be due to cell type, sequence variability. Thus it is recommended that the analysis of the methylation profile should be performed on a larger cohort and combined with transcriptomics (from the same cells tested for epigenetic changes) as an improvement of interpretability.

### 1.3 Histone modifications

**FIGURE 3:**
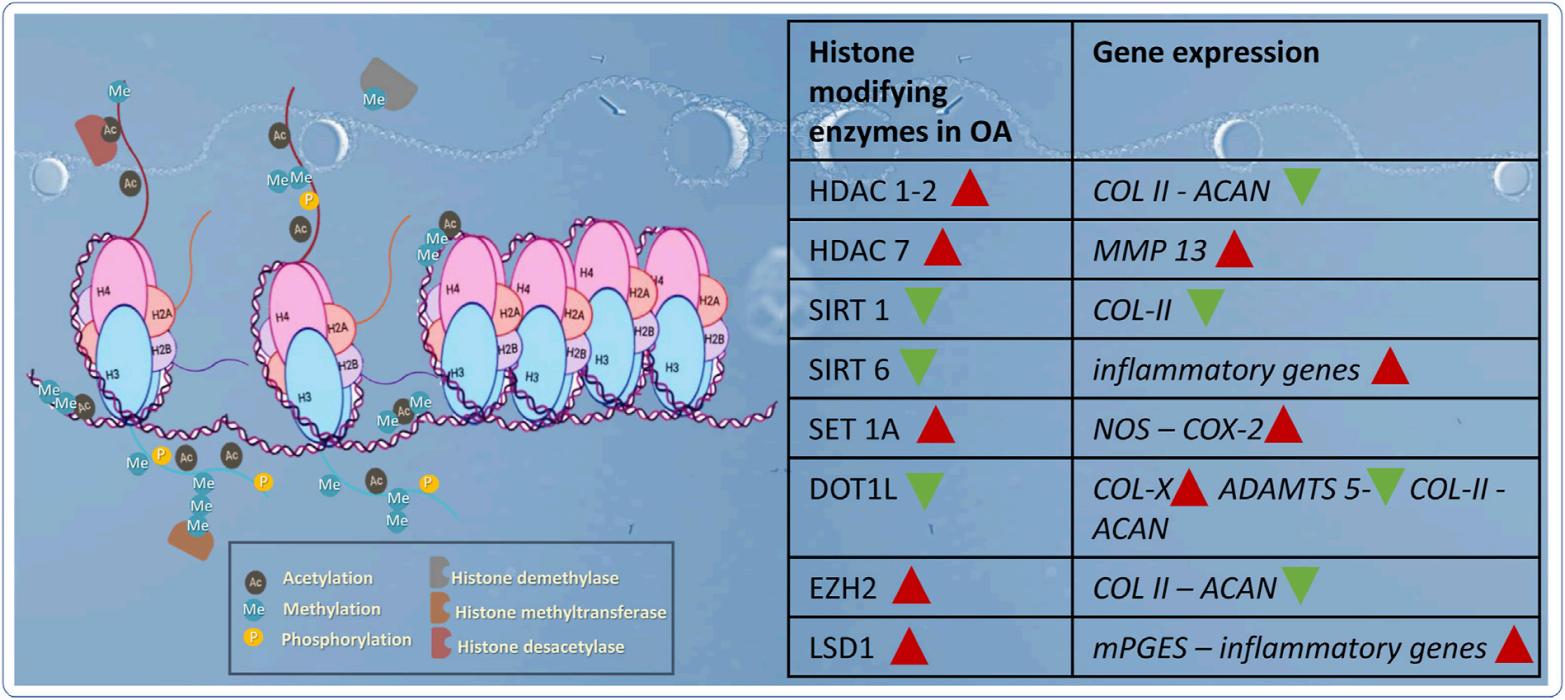
The key players of histone modifications in cartilage and the effects on targeted genes. Red arrows indicate catabolic genes are stimulated by the corresponding enzyme, whereas green arrows indicate anabolic genes are inhibited.

The amino-terminal residues of histones as well as carboxy-terminal of H2A and H2B histones are of post-translational changes such as acetylation, and methylation which are the most studied modifications (Figure 3). But there are nearly twenty modifications including phosphorylation, ubiquinitylation, sumoylation, O-GlcNAcylation, hydroxylation, formylation, ADP ribosylation, citrullination, succinylation, propionylation, butyrylation, 2-hydroxyisobutyrylation, and the most recent crotonylation (Huang et al., 2015).

Depending on the type of modification and the residue affected, these marks, which are reversible and dynamic, are established or removed by specific enzymes. These covalent changes play an essential role in regulating gene transcription by being closely associated with the state of chromatin. To date, the most documented histone modifications in the articular cartilage are the histone acetylation and the methylation.

#### 1.3.1 Histone acetylation

Acetylation of histones occurs mainly at lysine residues. This dynamic process is ensured by the action of antagonistic enzymes: Histone acetyltransferases (HAT) that add an acetyl group, and histone deacetylases (HDAC) that remove an acetyl group. HATs use the acetyl-CoA cofactor to catalyze the transfer of acetyl cluster to an amine group of a lysine. On the contrary, the removal of the acetyl group is carried out using the cofactor Zn^2+^ for the majority of HDACs. It may also be dependent on NAD^−^ when performed by sirtuins (a family consisting of seven members SIRT1-7), a specific class of HDACs. In general, HDACs have a low substrate specificity and are thus able to remove acetyl group at multiple sites. HATs and HDACs are very often associated with protein complexes that allow them to be recruited to specific loci and regulate their activity.

The presence of acetylated lysine is generally associated with transcription activation, while the presence of deacetylated lysine is associated with the suppression of transcription. Since acetyl group carries a negative charge, the acetylation of lysines neutralizes the positive charges of histones, which by electrostatic bonds interacts with negative DNA charges. Thus, the interaction between histones and DNA is reduced, increasing the accessibility of DNA to transcription factors and RNA polymerase II (Mizzen and Allis, 1998).

In addition to the direct effect of acetylation on the degree of chromatin compaction, acetylated lysines allow the recruitment of factors since they are recognized by proteins with a PHD domain (Plant HomeoDomain) or a bromodomain (Yang and Seto, 2007). Among bromodomain proteins, ATP-dependent remodeling complexes such as SWI2/SNF2, transcription factors, PAHs such as p300 or Gcn5, and TAFII 250 protein involved in transcription initiation have been identified. These proteins will in turn interact with other protein complexes to allow the release of chromatin. Beyond histones, other proteins can be acetylated by HATs. The tumor suppressor protein p53 was the first to be identified (Gu and Roeder, 1997). These reversible modifications in non-histone proteins play a key role in transcription activation, protein-protein interactions, stability regulation and DNA affinity (Singh et al., 2010).

#### 1.3.2 Histone methylation

Histone methylation can occur on both lysine and arginine residues. Like acetylation, histone methylation is a reversible and dynamic process that requires two types of enzymes: The histone methyltransferases (HMT) that add a methyl group and the histone demethylases (HDM) (Bannister and Kouzarides, 2011). Like DNMT, HDMs use the SAM donor to transfer the methyl cluster to the lateral lysine chain group or the lateral arginine chain group. Unlike acetylation, methylation does not alter histone charge and therefore has no direct effect on chromatin remodeling. However, methylated residues are binding sites for different protein complexes that alter the structure of chromatin to influence transcription. Methylated residues can be recognized mainly by the ADD or the Tudor domain (Table 2). In addition, the interpretation of histone methylation is more complex since lysine may be mono-, di- or tri-methylated (noted me1, me2 or me3) and single- or di-methylated on arginine. Moreover, non-histone proteins can also be methylated to certain lysine or arginine residues.

**TABLE 2:** Recognition domains of methylated histones.

Modification	Position		Recognition domain	Example of protein	Function
Methylation of lysines	H3	K4me0	PHD	BHC80	Induces the association of LSD1 in promoter region
			WD40	WDR5/8	Allows the recruitment of methyltransferases
			ADD	DNMT3L	DNA methylation
		K4me	Chromo	CHD1	Chromatin remodeling
			PHD	PHF2/8	H3K9 demethylation
			Tudor	JMJD2A	Histone demethylase
			MBT	PHF20L1	—
			Zf-CW	ZCWPW1	—
		K9	Chromo	HP1	Heterochromatin
			PHD	SMCX	Demethylation
			Tudor	UHRF1	DNA methylation
			WD40	EED	Propagation of H3K27me
			Ankyrin	G9a/GLP	Methyltransferase
			BAH	BAH	Hetérochromatin
		K27	WD40	EED	Repression mediated by PRC
			Chromo	CBX7	Repression mediated by PRC
		K36	Chromo	MRG15	RNA splicing
			PWWP	DNMT3A	Guidance of DNA methylation
		K79	Tudor	53BP1	Response to double strand breaking
	H4	K20	Tudor	PHF20	—
			MBT	Sfmbt	Repression by the polycomb proteins
			PWWP	Pdp1	Induction of K20me3
			WD40	LRWD1	Replication of DNA
Methylation of arginines	H3	R17me2 asymetric	Tudor	TDRD3	Activation of transcription
	H4	R3me2 asymetric	Tudor	TDRD3	Activation of transcription
		R3me2 symetric	ADD	DNMT3A	—

In human, the methylated sites are lysines 4, 9, 27, 36, and 79 of histone H3 and lysine 20 of histone H4. Lysine methylation may be associated with repressive or activating (Bannister and Kouzarides, 2011). The functional consequence of such a change is dictated by the context, position and degrees of methylation. Generally, transcriptionally active promoters are enriched with H3K4me3, while transcriptionally repressed promoters are enriched with H3K9me3 and H3K27me3. Methylations of H3K36 and H3K79 are associated with transcription elongation.

Arginine methylation can be catalysed by two classes of arginine methyltransferases (type I and II PRMT) and is associated with either an activation or repression. Whether this type of methylation is reversible is still controversial.

##### 1.3.2.1 The methyltransferases (KMT)

Currently, more than 50 lysine methyltransferases have been identified. The mechanism of methylation by these enzymes is almost identical because most of them contain the catalytic domain SET (su (var)3-9, enhancer-of-zest, trithorax) preceded and followed by areas rich in cysteine that are necessary for methyltransferase (Herz et al., 2013). To date, only the enzyme DOT1L, which methylates the globular region of the H3 histone at the K79 level, does not contain the SET domain but has a catalytic domain structurally close to PRMT (Nguyen and Zhang, 2011). Despite this, the methylation mechanism is very close to other KMTs. Based on the sequence of their catalytic site and adjacent protein regions, KMTs containing a SET domain can be separated into four classes (Morera et al., 2016): SET1 (including EZH1 and EZH2), SET2 (containing NSD1-3, SETD2 and members of the SMYD family), SUV39 (SUV39H1, SUV39H2, G9a, GLP, ESET and CLLL8) and RIZ (for “retinoblastoma-interacting zinc finger” including RIZ1, BLIMP1, PFM1).

##### 1.3.2.2 The lysine demethylases (KDM)

The 30-member lysine demethylases are divided into two families: The KDM1 family (KDM1A and KDM1B, also known as Lysine Specific Demethylase 1 and 2, LSD1 and LSD2, respectively) and the family containing the Jumonji C domain (JmjC) (Shi et al., 2004; Tsukada et al., 2005). Using the FAD cofactor (Flavine Adenine Dinucleotide), the KDM1 family is able to demethylate mono-and di-methylation through their amine oxidative activity (Shi et al., 2004). Unlike this family, the JmjC family is also able to demethylate trimethylation by hydroxylation and using Fe^2+^ ion and 2-oxoglutarate (or *α*-ketoglutarate) as a cofactor (Dong et al., 2014). With about 30 members, the JmjC family can be separated into seven subfamilies named KDM2 at KDM8. These enzymes have a high specificity and specifically target a residue and/or a methylation level. For example, the SET7 enzyme only generates the HEK4me1 mark.

##### 1.3.2.2 Interrelation with other epigenetic modifications

It has been observed that the post-translational modifications of histones can influence the recruitment of DNMT at specific loci in order to coordinate the different epigenetic marks such as the DNA methylation. Thus, the epigenetic status of tail residues of histones plays a critical role in establishing DNA methylation as illustrated by the specific recognition of the non-methylated H3K4 mark by the DNMT3L (Ooi et al., 2007).

Conversely, DNA methylation is also able to guide the modification of histones. All of these data underline the coordination and cooperation that exist between the various epigenetic factors. Altogether, these data point out that various epigenetic factors can be coordinated and do cooperate. These interactions allow the establishment of a specific epigenetic signature of a chromatin state contributing to distinct biological mechanisms.

### 1.4 Modifying the histone marks to prevent osteoarthritis

In recent years, the study of histone modification has emerged as a new field in the context of osteoarthritis. Several studies have reported a role of these epigenetic marks in the synthesis and degradation of cartilage and have been listed in the Figure 3. To date, histone acetylation is the most studied modification in articular cartilage although recently, some studies have focused on the methylation of histones.

#### 1.4.1 Modifying the acetylation of histones

Numerous studies have shown that HDAC inhibitors provide a chondroprotective role in modulating the expression of genes encoding MMPs and matrix components (Young et al., 2005; Wang et al., 2009a; Kim et al., 2015). In a model of osteoarthritis obtained by the destabilisation of the medial meniscus in mice, it has been shown that the administration of trichostatin A, a large HDAC inhibitor, significantly reduces cartilage damage in both the tibial plateau and the femoral condyle (Culley et al., 2013).

##### 1.4.1.1 The HDACs and OA

Several HDACs appear to be involved in the regulation of specific cartilage genes (Table 3). Expression of the HDAC7 enzyme has been shown to increase in osteoarthritic cartilage and contribute to the activation of transcription of the *MMP-13* (Higashiyama et al., 2010). In combination with the transcription factor Snail, the enzymes HDAC1 and HDAC2, whose expression is also elevated in OA chondrocytes, suppress the expression of *COL2A1* and *ACAN* genes*.* HDAC1 also inhibits the expression of *COL9A1* while HDAC2 inhibits the expression of *COMP* and *COL11A1* (Hong et al., 2009). Finally, the HDAC4 enzyme, which plays a crucial role during skeletogenesis by inhibiting *Runx2* (Vega et al., 2004), notably as a target of PTHrP, also appears to be involved in the pathogenesis of osteoarthritis with different effects depending on the stage of the pathology. Indeed, one study reported a decrease in HDAC4 expression in OA cartilage compared to healthy (Cao et al., 2014), while two other studies observed the opposite (Lu et al., 2014; Song et al., 2015). In the first study, decreased HDAC4 expression is associated with an increase in *Runx2* and *MMP13* since HDAC4 decreases the promoter activity of these two genes (Cao et al., 2014). At the same time, HDAC4 partially blocks the effect of IL-1 on the expression of catabolic genes such as *iNOS, COX-2, ADAMTS-4* and *-5.* In contrast, Lu and his colleagues have shown that HDAC4 invalidation on a human chondrosarcoma cell line SW1353 suppresses the expression of *MMP-1, -3, -13,* and *ADAMTS-4* but also the expression of several anabolic genes such as *ACAN* (Lu et al., 2014). Although in this study HDAC4 expression is increased in OA cartilage, the authors did report a negative correlation between HDAC4 expression and the severity of pathology. Thus, all of these data suggest a different expression and role of HDAC4 depending on the stage of osteoarthritis (Lu et al., 2014).

**TABLE 3:** List of enzymes involved in the modification of histones in human OA cartilage.

Enzyme	Expression in OA cartilage	Effects	References
Histone acetylation			
HDAC1	Increase	Repression of *COL2A1*, *ACAN*, *COL9A1*	Hong et al. (2009)
HDAC2	Increase	Repression of *COL2A1*, *ACAN*, *COMP*, *COL11A2*	Hong et al. (2009)
HDAC4	Variation depending on grade	Repression or stimulation of catabolism	Cao et al. (2014); Lu et al. (2014)
HDAC7	Increase	Induction of *MMP-13*	Higashiyama et al. (2010)
SIRT1	Decrease	Increase of *COL2A1*, *ACAN*	Dvir-Ginzberg et al. (2008); Gabay et al. (2012)
SIRT6	Decrease	Repression of inflammatory genes	Wu et al. (2015)
Histone methylation			
SET-1A	Increase	Induction of *iNOS* and *COX-2*	El Mansouri et al. (2011)
EZH2	Increase	Repression of *SFRP-1*	Chen et al. (2016)
LSD1	Increase	Induction of *mPGES-1*	El Mansouri et al. (2014)
DOT1L	Heterogenous	Repression of Wnt signaling	Monteagudo et al. (2017)
PRMT5	Increase	Repression of ECM molecules	Dong et al. (2022)

##### 1.4.1.2 The sirtuins and OA

In addition to its role in chondrocyte survival (Gagarina et al., 2010; Gabay et al., 2012; Oppenheimer et al., 2012), the histone deacetylase SIRT1 also appears to play a crucial role in cartilage homeostasis. Although this enzyme is generally associated with transcriptional suppression, SIRT1 promotes the expression of Sox9 target genes, including *COL2A1* and *ACAN,* in human articular chondrocytes (Dvir-Ginzberg et al., 2008). Consistent with these findings, SIRT1 protein expression is also decreased in osteoarthritic human cartilage compared to healthy cartilage (Dvir-Ginzberg et al., 2008). In addition, heterozygous mice for SIRT1 ± show more severe signs of osteoarthritis than SIRT1 +/+ mice with reduced expression of COL2A1 and ACAN (Singh et al., 2010). Similarly, surgically-induced osteoarthritis is accelerated in mice conditionally invalidated for SIRT1 in articular cartilage compared to wild type (Matsuzaki et al., 2014). It is only recently that the SIRT1-mediated mechanisms controlling the expression of COL2A1 and ACAN have been highlighted. It has been shown that SIRT1 forms a complex with the Sox9 transcription factor allowing its deacetylation. Although the removal of this post-translational modification does not affect Sox9 affinity on the promoter of the *COL2A1 gene*, it has been shown to promote Sox9 translocation in the nucleus leading to increased expression of the *ACAN* (Bar Oz et al., 2016). In addition, SIRT1 forms a complex with the KMT SET7/9 onto the promoter of *COL2A1.* SET7/9 has been shown to inhibit the deacetylase activity of SIRT1 and to methylate H3K4. At the same time, HAT p300 and GCN5 are recruited onto the same region, leading to lysine acetylation (Furumatsu et al., 2005; Oppenheimer et al., 2014). Altogether, these histone changes contribute to the activation of the transcription of *COL2A1*. Besides SIRT1, the presence of SIRT6, another enzyme belonging to the sirtuin family, was also shown to be reduced in OA chondrocytes compared to healthy ones. The protective effect of this enzyme on articular cartilage has been observed *in vivo* and *in vitro*: by its deacetylase activity, SIRT6 reduces the expression of inflammatory genes induced by NF-kB, and suppresses the senescence of chondrocytes (Wu et al., 2015).

#### 1.4.2 Modifying the methylation of histones

Although histone methylation is a less well-studied area in the cartilage context, it appears to be also implicated in the pathogenesis of osteoarthritis (Table 3).

##### 1.4.2.1 The lysine methyltransferases (KMT) and OA

The first direct link between this epigenetic mark and osteoarthritis was revealed in 2011. El Mansouri and his colleagues observed an increase in expression of KMT SET-1A in OA cartilage compared to healthy (El Mansouri et al., 2011). The team described that IL-1β triggers the recruitment of this enzyme onto the promoters of the iNOS and *COX-2 genes.* By di- and tri-methylating H3K4, SET-1A also activates the transcription of these two genes involved in chondrocyte apoptosis, the MMP synthesis, and reduces collagen production (El Mansouri et al., 2011). Another study investigated the modification of epigenetic marks within the promoter of Sox9 during hip OA (Kim et al., 2013): It was shown an increase of the repressive marks H3K9me3 and H3K27me3, and a decrease of the acetylation of H3K9, K15, K18, K23, and K27 that may explain the decrease in *Sox9* expression in this pathology. A new KMT has been shown to play a role in OA and more specifically in triggering the hypertrophy of chondrocytes. The enzyme EZH2 has been shown to be overexpressed in OA chondrocytes compared to healthy chondrocytes, probably mediated by IL-1 (Chen et al., 2016). EZH2 is recruited at the sFRP-1 gene, an inhibitor of the Wnt signaling, and tri-methylates H3K27 within the promoter and finally represses its expression. Thus, the overexpression of EZH2 activates the Wnt/- β catenin signaling, leading to the increase of *COL10, MMP13, ADAMTS-4* and -*5* gene expression. Intra-articular injection of an EZH2 inhibitor in an osteoarthritic mouse model, induced by the anterior cruciate ligament section, delays the development of osteoarthritis (Chen et al., 2016). It has been shown that the inhibition of EZH2 improves OA (Allas et al., 2020) through inhibiting the Wnt/beta catenin pathway. However, X. Du et al. (2020) showed conflicting data and reported a positive role for Ezh2 notably by inhibiting the chondrocyte hypertrophy. They identified an EZH2-positive subpopulation in OA patients which is responsible for regulating chondrocyte healing, suggesting EZH2 might act as a potential target for OA diagnosis and treatment.

DOT1L is another lysine methyltransferase potentially involved in cartilage homeostasis. It methylates the lysine 79 of histone H3 and is associated with OA: Its activity is downregulated in damaged area of OA patients compared to corresponding preserved area. In mice, its pharmacological inhibition triggers OA. It has been shown that DOT1L downregulates Wnt signaling and that the normal hypoxic signal within cartilage protects against OA.

As an arginine methyltransferase enzyme, PRMT5 was shown to be involved in the chondrogenesis. Moreover its expression is upregulated in the cartilage of patients with OA (Dong et al., 2020), and it was shown that its inhibition attenuates cartilage degradation in mice through the reduction of inflammatory signaling.

##### 1.4.2.2 The lysine demethylases (KDM) and OA

Despite the important role of LSD1 in the initiation and progression of several pathologies, only four studies have shown a role of LSD1 lysine demethylase in articular cartilage. In 2011, Rodova et al. (2011) studied the relationship between *NFAT1* (nuclear factor of activated T cells) and LSD1. The transcription factor *NFAT1* (also called *NFATC2*,) which was initially identified as a repressor of the immune response, could be involved in the pathogenesis of osteoarthritis. Indeed, *NFAT1 −/−* mice have cartilage histological characteristics similar to those observed in osteoarthritis in humans, including the cartilage degradation, the presence of osteophytes and clusters of chondrocytes (Wang et al., 2009b). Nevertheless, skeletal development appears to be normal at the histological level. To confirm these results at the cellular level, Rodova et al. (2011) have shown that NFAT1 plays an essential role in articular cartilage homeostasis only in adulthood, whereas its expression is weak in the embryo. Subsequently, the authors studied the regulation of this transcription factor by the demethylation of histones. Interestingly, they found that the expression of *NFAT1* is suppressed in the embryo (E16.5) by LSD1, *via* H3K4me2 demethylation, and activated in adults (6 months) by Jhdm2a, *via* H3K9me2 demethylation. The same team showed that LSD1 is also responsible for the decreased expression of *NFAT1* and *SOX9* in 18-month-old mice (Zhang et al., 2016c; Zhang et al., 2016d). LSD1 therefore plays a critical role in cartilage homeostasis. However, invalidation of LSD1 in these mice does not allow for increased expression of the target genes of NFAT1 and Sox9.

El Mansouri et al. (2014) studied the effect of LSD1 on its own on the expression of *mPGES1* (Microsomal prostaglandin E synthase-1) in human joint chondrocytes mPGES1 catalyzes the terminal stage of PGE2 biosynthesis, which mediates the inflammatory response contributing to the development of osteoarthritis. In this study, the authors showed that pro-inflammatory interleukin IL-1-induces the recruitment of LSD1 at the mPGES1 promoter level. LSD1 then demethylates H3K9me2 in order to activate the expression of this gene. This study highlights the involvement of LSD1 in the cartilage inflammation process and in the articular cartilage homeostasis.

Finally, the H3K27 demethylase UTX expression positively correlated with human knee OA and its forced expression aggravated the signs of OA (Lian et al., 2022). When invalidated in a chondrocyte-specific manner in mice, it was shown that the cartilage integrity was promoted suggesting that targeting the H3K27 methylation is in interesting approach for OA therapy.

## 2 Conclusion

The description of the DNA methylation changes occuring in pathological cartilage of cohorts of patients has been dramatically developped through Epigenome-Wide Association Studies (EWAS). These marks constitute an epigenetic profile as potential markers or predictors of OA. Classically, the experimental design is based on the comparison of individuals with phenotype (e.g., OA patients) with control subjects. But the interpretability of the EWAS is rather than complex. As illustrated in the Figure 2, OA is a progressive disease in which early and late stage should not be confounded. It can also affect various joints and tissues (not only the cartilage). This highlights the importance of considering very cautiously which cell type are compared although pathological individual samples are usually referred as “OA sample.” To what extent the epigenetic changes are causal or reflective of the observed phenotype is another concern: performing a transcriptomic study on the same cells tested for epigenetic changes should be considered. Finally, it should be kept in mind that a modest change of DNA methylation, although reproducible, may not necessarily make a great contribution to the mechanism of the disease and may hamper the mechanistic understanding.

Considering the various histone modifying enzymes involved in the regulation of key genes during OA, targeting these enzymes could be of therapeutic interest in the treatment of OA. It should be noted that many inhibitors of these enzymes are already approved by the United States Food and Drug Administration (FDA) or are under evaluation in clinical trial for the treatment of cancer or inflammation.

Regarding OA, several attempts have been performed in mouse: The pan-HDAC inhibitor Trichostatin A (TSA) shows an interesting reduction of cartilage loss in mouse OA model (Young et al., 2005). The HDAC inhibitors are of particular interest since the HDAC activity seems associated with catabolic metabolism and with the chondrocyte phenotype alteration. The expression of many relevant genes for the chondrocyte homeostasis (proinflammatory cytokines such as IL-1 or IL-6, extracellular matrix, or proteinases encoding genes) depends on their histone acetylation levels. Thus HAT inhibitors or HDAC inhibitors should be regarded as promising therapeutic options for OA. Non-etheless, conflicting effects were observed when HDAC inhibitors are added to chondrocytes depending on the time of exposure: A short-term treatment increases whereas a long-term treatment decreases the expression of collagen 2 and aggrecan.

Beside the acetylation modifiers, the inhibitors of histone methyl transferases, such as the EZH2 inhibitor (EPZ-6438) have been shown to protect articular cartilage from degradation in mice (Allas et al., 2020).

Similarly, the histone demethylase inhibitors such as GSK-J4 (an inhibitor of histone demethylase JMJD3 and KDM6A) are able not only to suppress the IL-1β induced production of proinflammatory cytokines and proteases, but also to prevent articular cartilage loss in a mouse model of OA (Jun et al., 2020). However, GSK-J4 inhibits *SOX9* and *COL2A1* expression during chondrogenesis of human MSCs. Thus these conflicting results suggest that the inhibition of histone demethylation could be used as a strategy for OA therapy, while the histone demethylation could be used to stimulate cartilage formation for tissue engineering purposes. This highlights that the genomic and transcriptional context seems critical when using an inhibitor. Moreover, it should be noted that histone proteins form a small fraction of the known methylated proteins. Thus the contribution of many non-histone protein methylation still remains to be evaluated when using these inhibitors in therapeutics.

As an alternative to chemical compound, peptide-based inhibitors, as analogues of H3K4 substrates playing a role of antagonist (e.g., inhibiting LSD1), or peptide competitors, which block the interaction of LSD1 with its transcription factors (leading to more selective activation of LSD1 target genes), are now being investigated in cancer therapies and could be also applied to OA. The use these peptides to functionalise biomaterials could be of great interest for tissue engineering purposes.

The results obtained in animal models demonstrate that these inhibitors are potential agents against OA and are essential tools to evaluate the global effect of inhibitors on the whole joint (made of several tissues and cell types which do not respond in the same way). Indeed, the histone modifying enzymes are not highly specific for a given gene, leading to various cell effects depending on the cell type and the genomic context in which the methylation occurs. These histone modifications are complex and involve many interactions of transcription factors, requiring coordination and cooperation between several epigenetic factors, which results in a global epigenetic signature. These intricate mechanisms must be intensively studied, delineated and finally integrated on the whole joint as a result. It is thus very likely that, rather than the single use of a given drug applied to any stage of OA, the therapeutic approach of one drug will require the clear delineation of which OA stage it should be used for. With this view, genetic models in which the inducible invalidation of a given histone modifying enzyme is obtained, can help understanding its precise contribution in the disease.
